# Yifeixiaoji decoction as an adjunct to osimertinib therapy in advanced EGFR-mutated NSCLC: protocol for a randomized, double-blind, placebo-controlled trial

**DOI:** 10.3389/fmed.2026.1890856

**Published:** 2026-08-18

**Authors:** Qiuting Feng, Changchang Sun, Jiangyang He, Yiyang Zhao, Chenbing Sun, Huirong Zhu, Ling Bi

**Affiliations:** 1 Department of Oncology, Yueyang Hospital of Integrated Traditional Chinese and Western Medicine, Shanghai University of Traditional Chinese Medicine, Shanghai, China; 2 Department of Medical Oncology, Shuguang Hospital, Shanghai University of Traditional Chinese Medicine, Shanghai, China; 3 Oncology Department of Traditional Chinese Medicine, The First Affiliated Hospital of Naval Medical University (Second Military Medical University), Shanghai, China

**Keywords:** traditional Chinese medicine, non-small cell lung cancer, epidermal growth factor receptor tyrosine kinase inhibitors, clinical trial, drug resistance

## Abstract

**Background:**

Although EGFR-TKIs have substantially improved outcomes in EGFR-mutated advanced NSCLC, acquired resistance ultimately leads to treatment failure in most patients. Yifeixiaoji Decoction (YFXJD), a traditional Chinese herbal formulation, has shown promise as an adjunctive strategy; however, evidence from prospective clinical studies for this specific formula remains limited. We therefore designed this trial to assess whether YFXJD could prolong progression-free survival when added to standard osimertinib therapy in advanced NSCLC.

**Methods:**

A total of 200 patients with stage IV EGFR-mutant NSCLC will be enrolled in a randomized, double-blind, placebo-controlled trial and allocated 1:1 to receive standard osimertinib therapy combined with either YFXJD granules or matched placebo. The primary endpoints is progression-free survival. Key secondary endpoints include change in TCM syndrome score, objective response rate, quality of life (EORTC QLQ-C30/LC13), safety (NCI CTCAE v5.0), and exploratory peripheral blood biomarker analyses.

**Expected results:**

This trial will provide prospective evidence regarding the efficacy and safety of YFXJD as an adjunct to osimertinib therapy in patients with advanced EGFR-mutant NSCLC and will determine whether this approach influences clinical outcomes.

**Clinical trial registration:**

https://itmctr.ccebtcm.org.cn, ITMCTR2025002306.

## Introduction

Epidermal growth factor receptor tyrosine kinase inhibitors (EGFR-TKIs) have substantially improved outcomes for patients with advanced non-small cell lung cancer (NSCLC) harboring sensitizing EGFR mutations, prolonging progression-free survival (PFS) and improving quality of life (1). NSCLC accounts for approximately 85% of all lung cancer cases, which remains the leading cause of cancer-related mortality worldwide (2). Despite these clinical benefits, acquired resistance to EGFR-TKIs develops in nearly all patients, with a median PFS typically ranging from 9 to 13 months for first-generation TKIs (3). Overcoming or delaying resistance has therefore become a major clinical challenge in this population.

Traditional Chinese medicine (TCM) may exert multi-targeted therapeutic effects, including modulation of immune function (4), symptom relief, improvement of quality of life, and potential influence on the tumor microenvironment (5). The CATLA trial, a multicenter randomized double-blind placebo-controlled study, demonstrated that adding Chinese herbal medicine to first-generation EGFR-TKIs (erlotinib, gefitinib, or icotinib) significantly prolonged median PFS from 10.94 to 13.50 months (HR 0.68, 95% CI 0.51–0.90, *p* = 0.0064) (6). The subsequent CATLA-2 protocol is currently evaluating Yiqi-Yangyin-Jiedu Decoction (YYJD) combined with osimertinib as first-line treatment (7). These studies provide important evidence for the potential of TCM as an adjunctive strategy but also highlight the need for formula-specific investigations (8).

Building on these findings and based on our preclinical observations that a combination of *Punica Granatum* Peel and Dioscorea Nipponica inhibited the activation of dormant lung cancer cells and suppressed tumor growth *in vivo* (9), our research team developed Yifeixiaoji Decoction (YFXJD). YFXJD is a multi-herb formulation containing specific components such as Dioscorea nipponica, *Punica granatum* pericarp, and *Houttuynia cordata*, which have demonstrated anti-dormancy and anti-tumor effects in preclinical models. Preliminary clinical observations suggested trends toward enhanced efficacy and reduced toxicity. However, prospective randomized controlled clinical evidence for this specific formula in the current era of uniform osimertinib therapy remains lacking.

To address this gap, we designed a single-center, randomized, double-blind, placebo-controlled clinical trial to evaluate whether adjunctive treatment with YFXJD can prolong PFS and improve clinical outcomes in patients with advanced EGFR-mutant NSCLC receiving standard osimertinib therapy. The study will also assess safety and explore the biological effects of YFXJD, providing evidence to guide the integrated use of Chinese and Western medicine in this patient population.

## Methods and analysis

### Study design

#### Trial design

This is a single-center, randomized, double-blind, placebo-controlled clinical trial with two parallel groups. A total of 200 patients with advanced EGFR-mutant NSCLC will be enrolled and randomly allocated in a 1:1 ratio to either the YFXJD group or the control group receiving placebo. Both groups will receive standard osimertinib therapy (80 mg orally once daily) throughout the study period.

#### Recruitment and informed consent

Participants will be recruited through outpatient and inpatient referrals and study advertisements at Shuguang Hospital, Shanghai University of Traditional Chinese Medicine. Eligible patients will undergo a structured interview during clinic visits or upon admission, during which the study procedures—including treatment duration, imaging schedule, blood sampling, potential risks, and participants’ rights to withdraw at any time—will be explained in detail. Written informed consent will be obtained prior to enrollment.

#### Allocation and intervention

After obtaining informed consent, participants will be randomized to receive either adjunctive YFXJD granules or matched placebo in addition to standard osimertinib therapy. The key components of YFXJD are presented in Table 1. Treatment will continue until predefined discontinuation criteria are met, including disease progression, intolerable adverse events, withdrawal of consent, or completion of the study protocol.

**Table 1:** Main components of YFXJD.

Chinese name	Latin name	Source plant	Amount (g)
Beishashen	Glehniae Radix	*Glehnia littoralis* F. Schmidt ex Miq.	12
Maidong	Ophiopogonis Radix	*Ophiopogon japonicus* (L. f.) Ker-Gawl.	9
Chuanshanlong	Dioscoreae Nipponicae Rhizoma	*Dioscorea nipponica* Makino	9
Mohanlian	Ecliptae Herba	*Eclipta prostrata* (L.) L.	9
Yuxingcao	Houttuyniae Herba	*Houttuynia cordata* Thunb.	9
Shiliupi	Granati Pericarpium	*Punica granatum* L.	9
Jiegeng	Platycodonis Radix	*Platycodon grandiflorus* (Jacq.) A. DC.	3

#### Follow-up and assessments

**Figure 1:**
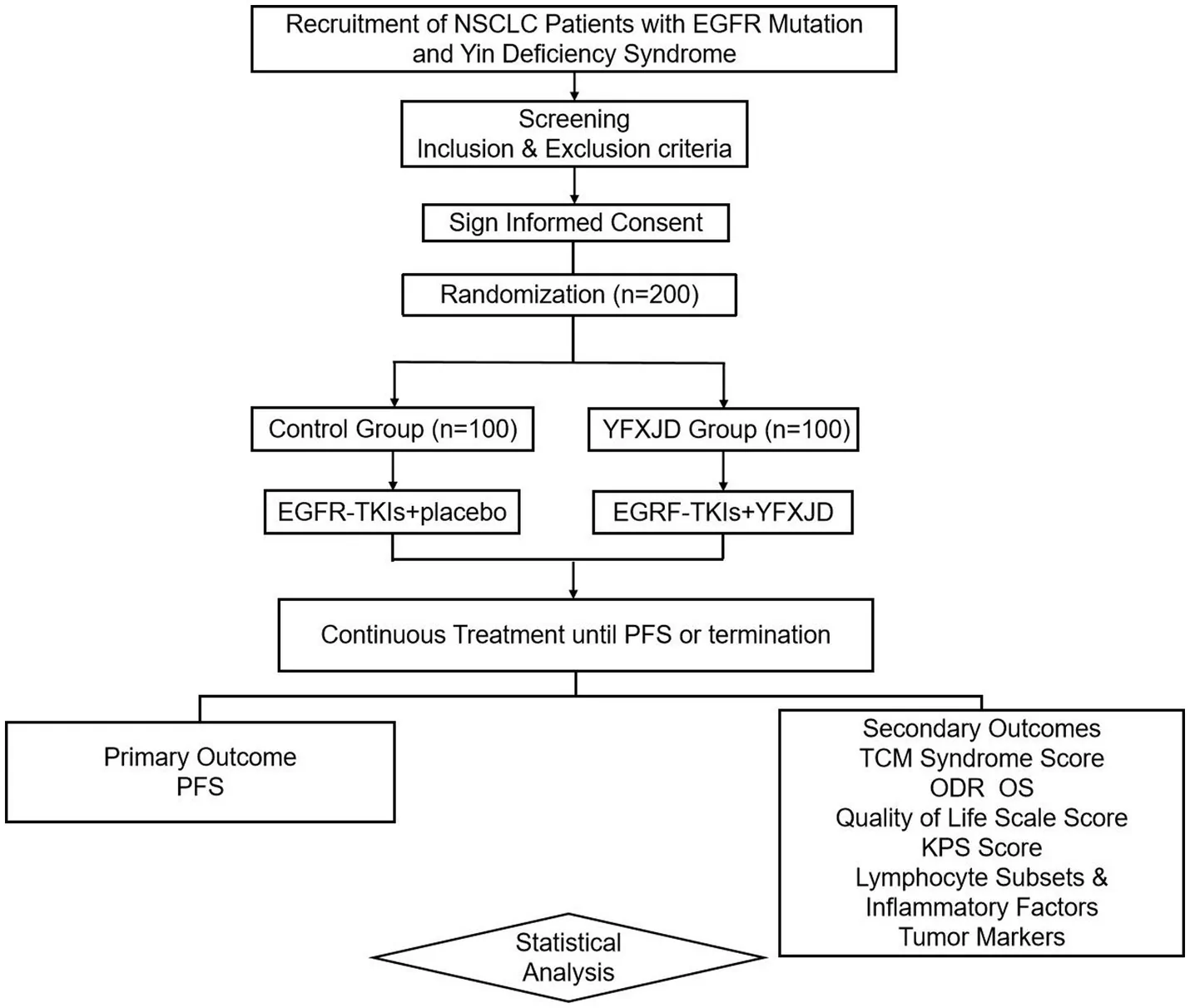
The flowchart of the trial.

Follow-up visits will occur every 3 months, as outlined in Table 2, and will include clinical evaluation, imaging studies, laboratory testing, and assessment of adverse events. Imaging for tumor response assessment will be performed every 8 weeks from randomization until disease progression. The efficacy of YFXJD will be evaluated through progression-free survival, TCM symptom scores, activity levels, biochemical parameters, and exploratory biological indicators. Safety will be assessed according to protocol-defined criteria. The trial flowchart is presented in Figure 1.

**Table 2:** Schedule of trial.

Assessment/Time point	Screening	Baseline/Initiation	Treatment period	Treatment period	Long-term follow-up	End-of-treatment visit	Survival follow-up
	D -7	D0	D90 ± 7	D180 ± 7	D365 ± 7	Upon PD/Discontinuation	
Procedures							
Informed consent	✓						
Eligibility review	✓						
Data collection							
Demographic information	✓						
History of NSCLC & treatment	✓						
History of other diseases	✓						
Randomization & drug dispensing		✓	✓ (Re-supply)	✓ (Re-supply)			
Primary endpoints							
1. PFS (RECIST 1.1)			Imaging per protocol	Imaging per protocol	Imaging per protocol	✓ (Confirm PD)	Record death
2. TCM Syndrome Score	✓ (Baseline)		✓ (Interim)	✓ (Primary Time Point)			
Secondary endpoints (Efficacy)							
Tumor response (ORR/DCR)			✓	✓	✓	✓ (Final)	
Quality of life (EORTC QLQ)	✓ (Baseline)		✓	✓	✓		
Performance status (KPS)	✓ (Baseline)		✓	✓	✓		
Biomarkers							
Lymphocyte subsets	✓ (Baseline)		✓	✓	✓		
Inflammatory cytokines	✓ (Baseline)		✓	✓	✓		
Tumor markers	✓ (Baseline)		✓	✓	✓		
Safety assessments							
Clinical laboratory tests							
CBC, hepatic & renal function	✓	✓	✓	✓	✓	✓	
Urinalysis & stool routine	✓	✓	✓	✓	✓	✓	
AE/SAE collection	✓	✓	✓	✓	✓	✓	
EGFR-TKIs specific AE recording		✓	✓	✓	✓	✓	
Concomitant medication record		✓	✓	✓	✓		
Proportion with dose Mod./Disc.			Recorded	Recorded	Recorded	✓ (Final)	
Study administration							
Protocol deviation record		✓	✓	✓	✓		
CRF completion		✓	✓	✓	✓	✓	

#### Ethics and registration

The study protocol was approved by the Medical Ethics Committee of Shuguang Hospital, Shanghai University of Traditional Chinese Medicine (approval number: 2025–1739–079-02) and registered with the International Traditional Medicine Clinical Trial Registry (ITMCTR2025002306) on April 12, 2025.

### Objectives

This study aims, through a randomized, double-blind, placebo-controlled trial, to evaluate the efficacy and safety of YFXJD combined with standard EGFR-TKIs therapy in NSCLC patients, explore its role in alleviating symptoms of TCM Qi-Yin deficiency, systematically assess the incidence and characteristics of treatment-related adverse events, and investigate its potential modulatory effects on serum inflammatory cytokines, peripheral blood lymphocyte subsets, and tumor markers.

### Participants

Participants will be recruited from patients with NSCLC who visited the outpatient clinic and inpatient ward of the Department of Oncology at Shanghai University of Traditional Chinese Medicine’s Shuguang Hospital. Diagnosis of NSCLC was confirmed through sputum and pleural effusion cytology examinations, bronchoscopy, lung biopsy, or histological examination of surgically resected tissue, in accordance with the 2022 edition of the “Guidelines for the Diagnosis and Treatment of Primary Lung Cancer” (10) issued by the National Health Commission. Clinical staging followed the 9th edition of the TNM classification system for lung cancer jointly published by the Union for International Cancer Control (UICC) and the American Joint Committee on Cancer (AJCC) in 2024 (11). Patients with clinical stage IV disease were selected. TCM diagnosis followed the criteria for Bronchopulmonary Carcinoma with Qi and Yin Deficiency Syndrome outlined in the 2016 “TCM Diagnosis and Treatment Guidelines for Malignant Tumors” (12) issued by the Tumor Professional Committee of the Chinese Association of Integrative Medicine: Primary Symptoms: Cough with little or no sputum, or scant frothy mucus; sputum may contain streaks of blood; five-heart heat (palms, soles, and chest); spontaneous sweating; fatigue. Secondary symptoms: Hoarseness, dry mouth and throat, insomnia; Tongue and pulse characteristics: Dry, cracked tongue with teeth marks, thin white or peeled coating, floating and rapid or deep, taut and fine pulse. A diagnosis is confirmed when two or more primary and secondary symptoms are present concurrently, combined with corresponding tongue and pulse findings.

### Inclusion criteria

Participants will be eligible if they meet all of the following criteria:

1. Histologically or cytologically confirmed diagnosis of NSCLC.
2. Radiologically confirmed stage IIIB, IIIC, or IV disease (according to the 9th edition of the AJCC Cancer Staging Manual) (11).
3. Presence of a sensitizing EGFR mutation confirmed by a validated molecular testing method (e.g., ARMS-PCR or NGS) from tumor tissue or plasma ctDNA (Primary EGFR-TKIs resistance mutations such as KRAS mutations and PTEN deletion are not included).
4. Planned to initiate first-line systemic therapy with osimertinib (80 mg orally once daily), without prior chemotherapy, immunotherapy, or definitive local therapy (including chemoradiotherapy) for the current disease.
5. Diagnosed with Qi-Yin Deficiency Syndrome according to TCM principles, as assessed by a certified TCM physician.
6. At least one measurable solid tumor per RECIST 1.1.
7. Aged 18 to 80 years (inclusive).
8. Karnofsky Performance Status (KPS) score ≥ 60.
9. Estimated life expectancy of at least 6 months.
10. Absolute neutrophil count (ANC) ≥ 1.5 × 10^9^/L; Platelet count ≥ 75 × 10^9^/L; Hemoglobin ≥ 90 g/L.
11. Total bilirubin ≤ 1.5 times the upper limit of normal (ULN); Alanine aminotransferase (ALT) and aspartate aminotransferase (AST) ≤ 2.5 x ULN (or ≤ 5 x ULN if liver metastases are present).
12. Serum creatinine ≤ 1.5 x ULN, or calculated creatinine clearance (CrCl) ≥ 50 mL/min (using the Cockcroft-Gault formula).
13. Willing and able to provide written, signed informed consent to participate in the study, and to comply with all study procedures and visit schedules.

### Exclusion criteria

Participants will be excluded if they meet any of the following criteria:

1. Known history of serious mental illness or cognitive impairment that, in the investigator‘s judgment, precludes the ability to provide informed consent or comply with the study procedures.
2. History of active alcohol or drug abuse within the past year.
3. Women who are pregnant, breastfeeding, or of childbearing potential who are unwilling to use a highly effective method of contraception during the study treatment period and for at least 3 months (or per specific EGFR-TKIs label requirement) there after.
4. Uncontrolled or clinically significant cardiovascular disease, including but not limited to: symptomatic congestive heart failure (NYHA Class ≥II), unstable angina, myocardial infarction within the past 6 months, or uncontrolled arrhythmias.
5. Poorly controlled hypertension (e.g., systolic blood pressure >150 mmHg or diastolic blood pressure >100 mmHg despite optimal medical management).
6. Active, uncontrolled systemic infection requiring intravenous therapy.
7. Any other severe, uncontrolled concomitant medical condition that, in the opinion of the investigator, would compromise the participant’s safety or interfere with the study evaluations.
8. Receipt of any systemic anti-cancer therapy (including chemotherapy, immunotherapy, other targeted therapy, or investigational agents) within 4 weeks prior to the first dose of study treatment.
9. Palliative radiotherapy to non-target lesions is permitted if completed >2 weeks prior to enrollment. Radiotherapy to target lesions or whole brain radiotherapy within 4 weeks prior to enrollment is excluded.
10. Prior definitive chemoradiotherapy for stage III disease.
11. Symptomatic, untreated, or unstable brain metastases or leptomeningeal disease requiring immediate local therapy (e.g., steroids, surgery, radiation). Participants with a history of treated brain metastases are eligible if they are radiologically stable (confirmed by MRI at least 4 weeks apart), asymptomatic, and do not require corticosteroid treatment for at least 2 weeks prior to the first dose.
12. History of another primary malignancy.
13. History of clinically significant interstitial lung disease (ILD), drug-induced ILD, or any evidence of active ILD on baseline imaging.
14. Known severe hypersensitivity to any component of YFXJD or to osimertinib.
15. Any condition that impairs oral drug absorption (e.g., inability to swallow, major gastrointestinal resection, active inflammatory bowel disease).

### Withdrawal/termination criteria

Participants will be withdrawn or terminated from the trial if any of the following conditions are met:

1. Failure to adhere to the key procedures outlined in the study protocol, which may compromise the validity of the data obtained from that participant.
2. The use of concomitant therapies that are prohibited by the protocol for the management of NSCLC or its symptoms.
3. Discontinuation of osimertinib due to toxicity: Occurrence of severe adverse reactions attributable to osimertinib therapy that necessitate the permanent discontinuation of the targeted agent, as determined by the treating physician. This criterion focuses on adverse events (e.g., severe interstitial lung disease, hepatotoxicity, or intolerable grade 3/4 rash or diarrhea) that make continued osimertinib treatment unsafe.
4. Withdrawal of consent: The participant voluntarily decides to withdraw from the study at any time for any reason.
5. Loss to follow-up: The participant becomes unreachable or fails to attend scheduled study visits without providing reason, despite repeated attempts at contact by the research team.
6. The principal investigator determines that continued participation is not in the best interest of the participant’s health and safety.

Participants who are discontinued from the study intervention will be encouraged to continue in the follow-up phase for the collection of survival and safety data, where feasible and with their consent. All reasons for withdrawal or discontinuation will be documented in the case report form.

### Interventions

#### Osimertinib intervention

According to the CSCO Guidelines for the Diagnosis and Treatment of Non-Small Cell Lung Cancer (2024) and the FLAURA trial (13), the recommended first-line osimertinib regimen for advanced EGFR-mutant NSCLC is 80 mg orally once daily, which can be taken with or without food (14). Treatment will be continued until disease progression, unacceptable toxicity, or other discontinuation criteria are met.

#### TCM intervention

The TCM intervention will be administered concurrently with EGFR-TKIs therapy and will continue until disease progression, unacceptable toxicity, or other criteria for treatment discontinuation are met. Both the YFXJD and placebo are manufactured as granules. Each sealed packet contains a fixed dose of granules (10 g per packet, 5:1 extraction ratio), with identical packaging to ensure complete blinding in terms of appearance, texture, smell, and taste. Matching placebo granules with similar appearance, odor, taste, and packaging, without active herbal ingredients. Are composed of pharmaceutical-grade excipients (primarily maltodextrin) with approved food colorants and flavorings.

Quality control of YFXJD granules includes: (1) botanical authentication of all raw herbs with voucher specimens deposited at the Shanghai University of TCM Herbarium; (2) compliance with Chinese Pharmacopoeia 2020 edition standards; (3) chemical fingerprinting by HPLC for batch-to-batch consistency; (4) 12-month stability testing; and (5) testing for pesticides, heavy metals, microbes, and adulterants. The YFXJD granules and placebo are produced under Good Manufacturing Practice (GMP) standards at Sichuan Neo-green Pharmaceutical Technology Development Co., Ltd. The manufacturer supplied the study products but had no role in study design, data collection, analysis, or manuscript preparation; no financial or non-financial competing interest exists.

Patients will be instructed to dissolve the contents of one packet in approximately 150–200 mL of hot water and to take it orally twice daily (morning and evening), 30 min after meals. The daily total dose will therefore consist of two packets. The dosage and administration frequency will remain constant throughout the treatment period unless modified due to tolerability issues as assessed by the investigator.

##### Adherence monitoring

Patients will return empty packets at each visit. Adherence will be calculated as (packets consumed / packets dispensed) × 100%, with ≥80% defined as adherent. Dose intensity, treatment interruptions, and modification rules will be recorded.

##### Blinding assessment

A blinding success questionnaire will be administered at the end of treatment to assess whether participants and investigators could correctly guess treatment assignment.

##### Herb-drug interaction review

Osimertinib is primarily metabolized by hepatic CYP3A4. The following measures will be implemented: (1) systematic review of all herbal components for potential CYP3A4 interactions; (2) prohibition of concomitant herbal medicines known to interact with CYP3A4 (e.g., St. John’s Wort, Glycyrrhiza, Scutellaria); (3) specific monitoring for overlapping hepatic toxicity (ALT/AST every 4 weeks), gastrointestinal toxicity, and pulmonary toxicity (ILD monitoring); and (4) dose modification guidelines for osimertinib in case of grade ≥3 toxicity.

Participants who experience severe adverse events (AEs) assessed as related to the trial intervention will be provided with necessary post-trial medical care and may receive appropriate compensation in accordance with relevant regulations and the insurance protocol for this clinical trial. For any adverse reactions determined to be unrelated to the study medication, standard medical management will be provided based on a comprehensive assessment of the participant’s condition, including medical history, clinical symptoms, physical signs, and results from laboratory tests, imaging, and other diagnostic examinations.

#### Concomitant treatment regulations

During the trial, participants will be prohibited from using any anti-tumor or traditional Chinese medicine interventions that could affect the evaluation of efficacy or safety. Medications for coexisting chronic conditions that require ongoing treatment must be permitted but documented in the case report form (CRF).

### Outcomes

#### Primary outcomes

Progression-Free Survival (PFS): Defined as the time from randomization to the first occurrence of radiographic disease progression according to RECIST 1.1 (15) criteria or death from any cause, whichever occurs first. Imaging will be performed every 8 weeks from randomization until disease progression, with blinded independent central review.

#### Secondary outcomes

1. Change in TCM Qi-Yin Deficiency Syndrome Score: The absolute change in the total score from baseline to 6 months (D180 ± 7), assessed using a pre-specified and validated TCM syndrome scale specific for Qi-Yin deficiency in lung cancer. The scale ranges from 0 to 48, with higher scores indicating more severe symptoms. The minimal clinically important difference (MCID) is 4 points. All TCM assessors will undergo a 2-day training session with standardized patients, and inter-rater reliability (kappa ≥ 0.80) will be established prior to study initiation.
2. Objective Response Rate (ORR) and Disease Control Rate (DCR) as per RECIST 1.1 (15).
3. Duration of Response (DOR).
4. Overall Survival (OS).
5. Change in health-related quality of life assessed using the European Organization for Research and Treatment of Cancer Quality of Life Questionnaire Core 30 (EORTC QLQ-C30) (16) and its lung cancer-specific module (QLQ-LC13) (17).
6. Change in Karnofsky Performance Status (KPS) score (18).
7. Incidence, type, severity, and relationship to study treatment of all Adverse Events (AEs) and Serious Adverse Events (SAEs), graded according to the National Cancer Institute Common Terminology Criteria for Adverse Events (NCI CTCAE) Version 5.0 (19).

Evaluation of EGFR-TKIs-related specific adverse reactions:

1. Rash: A validated skin toxicity assessment tool (e.g., HFSR-14 scale or specialized assessment based on CTCAE v5.0 dermatology criteria) will be used to record the incidence, maximum severity (grade), time to onset, duration, and management measures (e.g., topical/systemic medications, dose modifications) of rash.
2. Diarrhea: The incidence, maximum severity (graded per CTCAE v5.0 gastrointestinal criteria), duration, frequency of diarrhea will be recorded, along with the proportion of patients requiring EGFR-TKIs dose modification, interruption, or discontinuation due to diarrhea. The use of anti-diarrheal medications will also be documented.
3. The proportion of patients requiring EGFR-TKIs dose reduction, interruption, or permanent discontinuation due to the above or any other AEs.
4. Changes in serum levels of inflammatory cytokines (IL-2, IL-6, IL-10, TNF-*α*, IFN-*γ*).
5. Changes in peripheral blood lymphocyte subsets (CD3^+^, CD4^+^, CD8^+^, CD4^+^/CD8^+^ ratio, NK cells).
6. Changes in serum tumor marker levels (CEA, CYFRA21-1, SCC-Ag, NSE).

Biomarker sample processing: Blood samples will be collected at baseline, D90, D180, and D365. Samples will be centrifuged within 2 h of collection, aliquoted, and stored at −80 °C. Cytokines will be measured by ELISA, lymphocyte subsets by flow cytometry, and tumor markers by electrochemiluminescence.

Safety will be systematically monitored throughout the study and will include: Physical examinations and monitoring of vital signs at each study visit, with a focus on skin and gastrointestinal symptoms. Regular laboratory assessments: complete blood count, urinalysis, and blood chemistry panels (liver and renal function tests). Electrocardiograms (ECGs) at baseline and as clinically indicated. Ongoing and systematic surveillance, recording, and grading of all AEs according to NCI CTCAE v5.0 (19). Patients will be encouraged to use a standardized diary card to record daily details of skin rash and the frequency/severity of diarrhea episodes to enhance data collection accuracy.

### Randomization and blinding

To ensure balanced allocation, centralized randomization using an interactive web response system (IWRS) with variable block sizes (4 and 6) will be performed by an independent statistician. The randomization method will employ a 1:1 allocation ratio using SAS statistical software. Drug packages will be labeled with randomization codes; allocation codes will be sealed and stored separately. Participants will be enrolled sequentially by clinical staff and randomized via the IWRS. The treatment code will be retained by an independent statistician or pharmacy, not the principal investigators. All individuals involved in the trial—including clinicians, nurses, researchers, and patients—will remain unaware of the treatment assignments. Emergency unblinding will occur only when knowledge of assignment is essential for clinical management of a life-threatening condition; an SAE will not automatically trigger unblinding or withdrawal. Participants will be randomized before the first osimertinib dose. In the event of a life-threatening emergency, sealed emergency letters containing unblinding information will be available. A report of any serious AEs must be submitted to the principal investigators within 24 h. After the follow-up period ends, the treatment codes will be disclosed following the completion of data analysis.

### Sample size

The sample size for this study was calculated based on the primary outcome of progression-free survival (PFS). Based on the FLAURA trial, the median PFS for osimertinib monotherapy is approximately 18.9 months. To achieve 80% power at a two-sided significance level of α = 0.05 for detecting a clinically meaningful difference, a target hazard ratio (HR) of 0.66 was assumed, corresponding to a median PFS of approximately 28.6 months in the experimental group (an absolute extension of 9.7 months). This effect size is consistent with the HR observed in the CATLA trial (HR 0.68).

Using the Schoenfeld formula for the log-rank test, the required number of PFS events (d) is calculated as:

$$d=4\times {\left(Z\_(1-\alpha /2)+Z\_(1-\beta )\right)}^{2}/{\left(\ln\;\mathrm{HR}\right)}^{2}$$

With α = 0.05 (two-sided), β = 0.20 (power = 0.80), Z_(1-α/2) = 1.96, Z_(1-β) = 0.84, and HR = 0.66:

$$d=4\times {\left(1.96+0.84\right)}^{2}/{\left(\ln\;0.66\right)}^{2}\approx 176\;\text{events}$$

Under a 1:1 randomization ratio with an accrual period of 12 months, a minimum follow-up of 12 months, and an expected event rate of approximately 65% at the end of follow-up, the required total sample size is approximately 270 patients (135 per group). Accounting for an estimated dropout rate of approximately 20%, we plan to enroll 200 patients (100 per group) to ensure that sufficient patients complete the primary endpoint assessment. The planned enrollment of 200 patients is expected to yield approximately 130 PFS events, providing approximately 75% power at the planned analysis. An interim analysis for futility is planned after 50% of the expected PFS events have been observed, with the option to increase sample size if warranted by conditional power calculations.

### Data collection and management

Patient data will be prospectively collected using standardized Case Report Forms (CRFs) and entered into a secure REDCap electronic database. Baseline demographic and clinical characteristics, biomarker data, imaging-based tumor response, adverse events, concomitant medications, and biomarker samples will be recorded according to the study schedule (Table 2).

An independent Data Monitoring Committee (DMC), consisting of clinical epidemiologists, data monitors, and statisticians not involved in trial conduct, will review study safety, data completeness, and protocol deviations at regular intervals. The DMC charter specifies the composition, responsibilities, meeting schedule, and decision-making procedures. Based on these reviews, the DMC may recommend continuation, modification, or termination of the trial.

All source documents and biological samples will be stored securely and retained for at least 5 years after study completion in accordance with institutional and regulatory requirements.

Data sharing: Anonymized individual participant data will be shared upon reasonable request after publication, in accordance with applicable data protection regulations and participant consent.

Patient and public involvement: No patients or members of the public were involved in the design, conduct, or reporting of this trial.

### Statistical analysis

The statistical analysis will be performed using SAS statistical software (version 9.4). The primary analyses for efficacy and safety will be conducted according to the Intention-to-Treat (ITT) principle, including all randomized participants in the groups to which they were assigned. A per-protocol (PP) analysis will also be performed as a supplementary analysis.

#### Handling of missing data

For the primary endpoint (PFS), censoring will be applied at the last known follow-up date for patients without a progression event. For other continuous variables with missing values, multiple imputation will be used for primary analyses, with sensitivity analyses using Last Observation Carried Forward (LOCF).

#### Descriptive statistics

Continuous variables will be presented as mean ± standard deviation for normally distributed data or median (interquartile range) for non-normally distributed data. Categorical variables will be summarized as frequencies and percentages.

#### Comparative analyses

Between-group comparisons of continuous variables will use the independent samples t-test or Mann–Whitney U test, as appropriate. Categorical variables will be compared using the Chi-square test or Fisher’s exact test. For repeated measures (TCM syndrome score, QoL), baseline-adjusted mixed models for repeated measures (MMRM) will be used, with treatment group, visit, treatment-by-visit interaction, and baseline value as fixed effects, and participant as random effect.

#### Survival analysis

PFS and OS will be estimated using the Kaplan–Meier method, and groups will be compared with the log-rank test. The Cox proportional hazards model will be used to estimate hazard ratios (HRs) and corresponding 95% confidence intervals, with potential adjustment for prespecified stratification factors. Proportional hazards assumptions will be checked using Schoenfeld residuals.

### Additional analyses

Interim analysis for safety and futility is planned after 50% of the planned PFS events have been observed. A futility boundary will be defined using the Lan-DeMets O’Brien-Fleming spending function. The DMC will have clear decision authority regarding continuation, modification, or termination.

Subgroup analyses are prespecified based on factors such as EGFR mutation subtype (e.g., exon 19 del vs. L858R), baseline TCM syndrome score severity, and presence of brain metastases. These analyses are explicitly labeled as exploratory due to limited power for interaction testing.

Logistic regression analysis will be employed to explore factors influencing tumor response rates. A two-sided *p* < 0.05 will be considered statistically significant for the primary endpoint. No adjustment for multiple comparisons will be made for exploratory secondary endpoints, and results for these will be interpreted as hypothesis-generating.

#### Safety population

All randomized participants who received at least one dose of study treatment.

### Trial status

Participant enrollment commenced in January 2026 and is scheduled to conclude by December 2026. As of the revision date (July 2026), 38 patients have been enrolled. The final follow-up for the primary endpoint assessment is projected by December 2027. Database lock and final data analysis are planned for Q1 2028. This study protocol (Version 1.0, April 2025) was finalized prior to the initiation of participant recruitment. No amendments have been made since protocol version 1.0.

### Ethics and dissemination

This trial will be conducted in full conformity with the ethical principles outlined in the Declaration of Helsinki. The study design also adheres to the Standard Protocol Items: Recommendations for Interventional Trials (SPIRIT) 2025 Statement (20) and the SPIRIT-TCM Extension 2018 (21) for traditional Chinese medicine trials. The study protocol and informed consent forms have been reviewed and approved by the Medical Ethics Committee of Shuguang Hospital, affiliated with Shanghai University of Traditional Chinese Medicine (Approval No.: 2025–1739–079-02). Any subsequent amendments to the protocol will require submission and approval by the same ethics committee before implementation. Written informed consent will be obtained from each participant prior to any study-specific procedures. The trial is registered with the International Traditional Medicine Clinical Trial Registry (ITMCTR2025002306). Upon completion of the study, the results will be submitted for publication in peer-reviewed international scientific journals, regardless of the outcome. Findings will also be presented at relevant scientific conferences. Authorship eligibility will be determined based on the International Committee of Medical Journal Editors (ICMJE) criteria, reflecting substantial contributions to the work. Data sharing will be considered following publication, in accordance with applicable data protection regulations and participant consent.

## Discussion

Acquired resistance to EGFR-TKIs remains a major challenge in the management of advanced EGFR-mutant NSCLC, often developing within the first year of therapy. Although third-generation osimertinib has extended median PFS to approximately 18.9 months, resistance eventually develops in nearly all patients (22). Early adjunctive interventions that may modulate the internal environment and support patient resilience could provide an opportunity to delay the onset of resistance. This trial is designed to evaluate whether adding Yifeixiaoji Decoction (YFXJD) to standard osimertinib therapy can influence clinical outcomes when administered before resistance emerges.

YFXJD is a multi-herb TCM formula developed based on the principles of “supplementing qi, nourishing yin, detoxifying, and resolving masses.” Preclinical studies suggest that components of the formula, particularly Dioscorea nipponica and *Punica granatum* pericarp, may affect immune function, inflammatory responses, and tumor cell behavior *in vivo*, and preliminary clinical observations indicate potential benefits in symptom management and quality of life. These findings provide a rationale for investigating YFXJD in combination with osimertinib therapy, without presuming that it can reverse established resistance.

This study employs a randomized, double-blind, placebo-controlled design with two parallel groups, ensuring rigorous evaluation of YFXJD in a controlled setting. Key methodological strengths include: (1) the use of a single contemporary EGFR-TKI (osimertinib) to eliminate treatment heterogeneity; (2) blinded independent central review of radiographic endpoints; (3) a comprehensive biomarker panel to explore potential mechanisms; and (4) adherence to SPIRIT 2025 and SPIRIT-TCM 2018 reporting standards.

In addition to clinical endpoints, the trial incorporates exploratory biomarker assessments, including immune and inflammatory parameters, to provide mechanistic insights. These features strengthen the ability to generate reliable data regarding the safety and potential efficacy of the integrative approach.

Several limitations should be considered. As a single-center trial, the findings may have limited generalizability due to local clinical practices and participant characteristics. The complex composition of YFXJD and the limited scope of biomarker analysis constrain mechanistic understanding. Standardizing TCM syndrome differentiation within a clinical trial framework remains challenging. Fourth, the exploratory biomarker analyses are hypothesis-generating and require validation in future studies. Finally, although the use of osimertinib as a uniform background therapy addresses the heterogeneity concern raised by prior studies, the sample size may still be insufficient for robust subgroup analyses.

The CATLA trial (6) previously demonstrated that adding an unspecified Chinese herbal formula to first-generation EGFR-TKIs prolonged PFS. The ongoing CATLA-2 trial (7) is evaluating YYJD combined with osimertinib. Our trial complements these efforts by evaluating a distinct formula (YFXJD) with a different composition and preclinical rationale in a uniform osimertinib background. Positive results would provide evidence for a specific TCM formula that could be integrated into standard osimertinib therapy, while negative results would help refine the selection of herbal formulas for future investigation.

Future multi-center studies with larger sample sizes and more comprehensive mechanistic analyses—including serial ctDNA monitoring and paired tissue analysis at progression—will be necessary to confirm and extend these findings and to establish whether YFXJD specifically affects molecular resistance mechanisms.
